# Soybean isoflavones modulate gut microbiota to benefit the health weight and metabolism

**DOI:** 10.3389/fcimb.2022.1004765

**Published:** 2022-09-02

**Authors:** Lili Huang, Tao Zheng, Huaying Hui, Guozhen Xie

**Affiliations:** ^1^ College of Chinese Medicine, Hunan University of Chinese Medicine, Changsha, China; ^2^ School of Pharmacy, Hunan University of Chinese Medicine, Changsha, China

**Keywords:** soybean isoflavones, gut microbiota, enzyme activity, metabolism, short-chain fatty acids

## Abstract

Soybean isoflavones (SIs) are widely found in food and herbal medicines. Although the pharmacological activities of SIs have been widely reported, their effects on the intestinal microecology of normal hosts have received little attention. Five-week-old Kunming (KM) mice were administered SIs (10 mg/kg/day) for 15 days. Food intake, body weight, and digestive enzyme activity were measured. Small intestine microbiota, including lumen-associated bacteria (LAB) and mucosa-associated bacteria (MAB), were analyzed using 16S ribosomal ribonucleic acid (16S rRNA) gene sequencing. Short-chain fatty acids (SCFAs) were analyzed using gas chromatography-mass spectrometry (GC-MS). The results showed that the mice that consuming SIs showed a higher food intake but a lower body weight gain rate than that of normal mice. Sucrase, cellulase, and amylase activities reduced, while protease activity increased after SIs intervention. Moreover, SIs increased the intestinal bacterial diversity in both LAB and MAB of normal mice. The composition of LAB was more sensitive to SIs than those of MAB. *Lactobacillus*, *Adlercreutzia*, *Coprococcus*, *Ruminococcus*, *Butyricicoccus*, and *Desulfovibrio* were the differential bacteria among the LAB of mice treated with SIs. In addition, acetic acid, valeric acid, isobutyric acid, isovaleric acid, and caproic acid decreased, while butyric acid and propionic acid increased in the mice treated with SIs. Taken together, SIs are beneficial for weight control, even in short-term interventions. The specific mechanism is related to regulating the gut microbiota, changing digestive enzyme activities, and further affecting carbohydrate absorption and metabolism.

## Introduction

Soybean isoflavones (SIs) are mainly found in soy products (e.g., soybeans, soy milk, soy flour, and tofu) and Chinese material medica (e.g., *Pueraria*, *Fructus sophorae*, and *Philippine flemingia* root). Therefore, SIs are more commonly consumed in Asian than in western populations. It is well-known that SIs have estrogenic effects because their structures resemble 17-*β*-estradiol. Hence, SIs are considered phytoestrogens and have been applied as an alternative treatment for menopause-related symptoms (Chen and Chen, 2021). In addition, SIs are potential functional components with various health benefits, including anti-obesity (Luo et al., 2019), antioxidant (Li et al., 2020a), anti-inflammatory (Kim et al., 2019), and neuroprotective (Li et al., 2020b; de Rus Jacquet et al., 2021). The habitual intake of SIs may exert a potential prophylactic effect on metabolic syndrome in humans (Yamagata and Yamori, 2021). Although the health benefits of SIs have been widely reported, the mechanisms underlying their beneficial effects are not yet completely understood.

The intestine is the central organ for food digestion and absorption. Gut microbiota, inhabiting the intestine, participates in food metabolism by expressing numerous enzymes and is therefore considered a “metabolic organ” (Adak and Khan, 2019). Gut microbiota plays an important role in the metabolism of SIs. In general, SIs contain multiple components that are present in both the aglycone and glycoside forms. Among them, glycosides exhibit high polarity and poor bioavailability because of the saccharide group in their structure (Xie et al., 2022). The gut microbiota cuts the glycosidic bond in isoflavone glycosides and converts them into aglycones, which are more bioavailable than their parent compounds (Braune and Blaut, 2016; Landete et al., 2016; Koppel et al., 2017). It has been demonstrated that soybean isoflavone daidzin is converted to daidzein and equol by *Bifidobacterium* spp., *Eubacterium* spp., *Blautia* spp., and *Adlercreutzia* spp. (Braune and Blaut, 2016). Genistin, another soybean isoflavone, is converted into genistein by *Lactobacillus* spp., *Bacteroides* spp., and *Bifidobacterium* spp. (Steer et al., 2003). Daidzein, equol, and genistein exhibited greater estrogenic and antioxidant activities than those of daidzin and genistin (Gaya et al., 2017).

However, recent studies have demonstrated that SIs reduce the risk of intestinal diseases by enhancing intestinal secretion capacity, regulating inflammatory signaling pathways, and affecting intestinal barrier function and flora (Wu et al., 2020). SIs increased the proportion of *Lactobacillus* and reduced *Bacteroides* S24-7 and *Allobaculum* in type 2 diabetes mellitus (T2DM) mice (Yuan et al., 2021). Furthermore, it is currently believed that dietary supplementation with SIs is essential for improving intestinal homeostasis by mediating the growth of harmful bacteria such as *Klebsiella*, *Pseudomonas*, *Acinetobacter*, *Escherichia Shigella*, and *Staphylococcus*, as well as beneficial bacteria such as *Bifidobacterium*, *Bacteroides*, *Dorea*, *Faebacterium*, *Lactobacillus*, and *Weissella* (Al-Nakkash and Kubinski, 2020; Chen et al., 2022). Therefore, the gut microbiota is considered an important target for SIs in healthy and therapeutic diseases.

Previous studies have primarily focused on the anti-inflammatory, lipid-lowering, and inhibitory effects of SIs. However, little information is available regarding the effects of SIs on the gut microbiota of healthy hosts. Thus, in this study, we aimed to explore the healthcare potential of SIs in a normal host by investigating their regulating effects on the gut microbiota and metabolism.

## Materials and methods

### Reagents

Sodium chloride (NaCl) and foline-phenol (C_6_H_6_O) were purchased from Tianjin Fengchuan Chemical Reagent Co., Ltd. Potassium dihydrogen phosphate (KH_2_PO_4_) and 3,5-dinitrosalicylic acid (DNS) were purchased from Sinopharm Chemical Reagent Co., Ltd. Disodium hydrogen phosphate (Na_2_HPO_4_) was purchased from Tianjin Hengxing Chemical Reagent Manufacturing Co., Ltd. SIs (H05M9R54983), composed of daidzin (57.98%), glycitin (23.09%), genistin (6.92%), daidzein (2.12%), glycitein (0.74%), and genistein (0.4%), were purchased from Shanghai Yuanye Biotechnology Co., Ltd.

Reference standards, including acetic acid (≥ 99.5%), propionic acid (≥ 99.0%), isobutyric acid (≥ 99.0%), butyric acid (≥ 99.0%), isovaleric acid (≥ 99.0%), valeric acid (≥ 98%), caproic acid (≥ 99.5%), and isocaproic acid (≥ 98%), were purchased from Sigma-Aldrich (Merck, Darmstadt, Germany).

### Animal experiments

Specific pathogen-free (SPF) male Kunming (KM) mice (five-week-old), with license number SCXK (Xiang) 2019-0004, were provided by the Hunan Slaccas Jingda Laboratory Animal Co., Ltd. The mice were housed in a room with standard temperature (23-25°C) and humidity (50-70%). Food and purified water were provided by the Animal Experiment Center of Hunan University of Chinese Medicine. The experiments were approved by the Animal Care and Use Committee of Hunan University of Chinese Medicine (LL2020102103).

**Figure 1:**
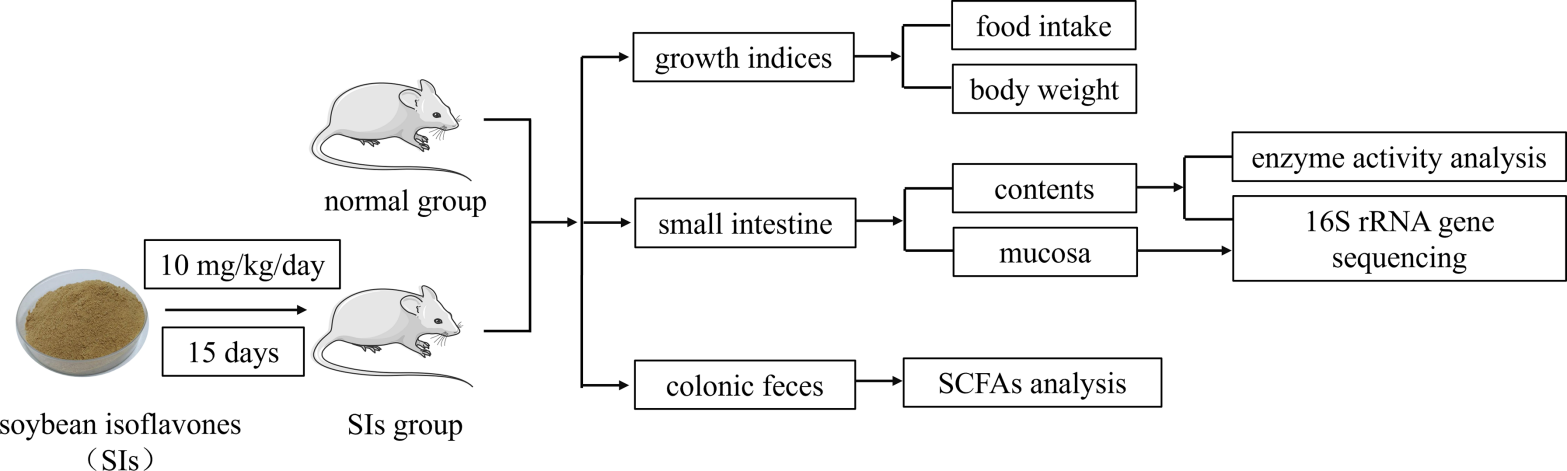
Experimental flow chart.

After three days of acclimation, the mice were randomly divided into normal (N) and SIs groups, with nine mice in each group. Mice in the SIs group were gavaged with SIs (10 mg/kg, 0.35 mL) once a day for 15 days (Jeon et al., 2020), whereas mice in the N group were given an equal amount of sterile water. Body weight was measured daily, and the food intake of each group was recorded every three days. Experimental flow chart was shown in **Figure 1**.

### Digestive enzyme activity analysis

After 15 days of intervention, mice were sacrificed by cervical dislocation. The content of the small intestine (from duodenum to ileum), from three mice in each group, was extruded using sterile tweezers and transferred into centrifuge tubes containing glass pearls and 20 mL of sterile water. The centrifuge tubes were shaken on a vortex mixer for 10 min and centrifuged at 3000 rpm for 15 min. The supernatant was collected and used for enzyme activity analysis (Tang et al., 2020). The activities of cellulase, amylase and sucrase were detected using the DNS colorimetric method (Zhao et al., 2013; Hui et al., 2018), and protease activity was measured using the foline-phenol method (Sun et al., 2018).

### 16S ribosomal ribonucleic acid (16S rRNA) gene sequencing

The small intestine contents (six mice in each group) were collected using the aforementioned method. After collecting the contents, the small intestine was gently rinsed with saline three times and then longitudinally cut with sterile scissors. The mucosa of the small intestine was collected using sterile coverslips (He et al., 2017; Long et al., 2017). Total genomic deoxyribonucleic acid (DNA) of the intestinal content and mucosa samples was extracted using the cetyltrimethylammonium bromide (CTAB) method. After DNA concentration and purity, the V3-V4 variable regions of the 16S rRNA genes were amplified using primers 341F (5’-CCTAYGGGRBGCASCAG-3’) and 806R (5’-GGACTACHVGGGTWTCTAAT-3’). The Illumina NovaSeq platform (Illumina, San Diego, CA, USA) was used for amplicon pyrosequencing. The bacteria in the intestinal content and mucosa were represented by lumen-associated bacteria (LAB) and mucosa-associated bacteria (MAB), respectively. The data presented in the study are deposited in the NCBI repository, accession number PRJNA854044.

### Short-chain fatty acids (SCFAs) analysis

Colonic feces were collected, frozen in liquid nitrogen, and thawed at room temperature before preparation. Phosphoric acid (50 μL, 15%), 100 mg glass beads, 100 μL 4-methylpentanoic acid (125 μg/mL), and 400 μL diethyl ether were added to each fecal sample, ground twice for 60 s at 60 Hz, and then centrifuged at 12000 rpm for 10 min. The supernatant was used for the analysis. After methodological investigation (**Supplementary Figure S1** and **Table S1**), the SCFAs contents were detected using a Thermo TRACE 1310-ISQ gas chromatography-mass spectrometer (GC-MS) (Thermo Fisher Scientific, Waltham, MA, USA).

### Bioinformatics and statistical analysis

USEARCH (version 10.0) was used to cluster the high-quality sequences at a similarity level of 97% to obtain operational taxonomic units (OTUs). The species taxonomy was annotated using the Greengenes database (Release 13_8). Alpha diversity indices, such as Chao1, Faith’s phylogenetic diversity (Faith_pd), Shannon, and Simpson were calculated using Quantitative Insights into Microbial Ecology version 2 (QIIME 2). The beta diversity distance was calculated using Bray-Curtis and visualized *via* principal coordinate analysis (PCoA). Linear discriminant analysis effect size (LEfSe) and DESeq2 were used to identify differential bacteria in the two groups.

Quality control and standardization of SCFAs data were performed using the R package “MetaboAnalystR” (**Supplementary Figures S2**, **3**). Orthogonal partial least squares-discriminant analysis (OPLS-DA) and support vector machines (SVM) were used to analyze SCFAs data. Kruskal-Wallis tests were used to analyze significant differences between the two groups. Redundancy analysis (RDA) was used for analyzing the interaction between differential bacteria and SCFAs metabolism. Statistical analysis was performed using unpaired *t*-test and data are expressed as mean ± standard deviation (SD), with *P* < 0.05 indicating significance.

## Results

### Effects of SIs on the growth of normal mice

**Figure 2:**
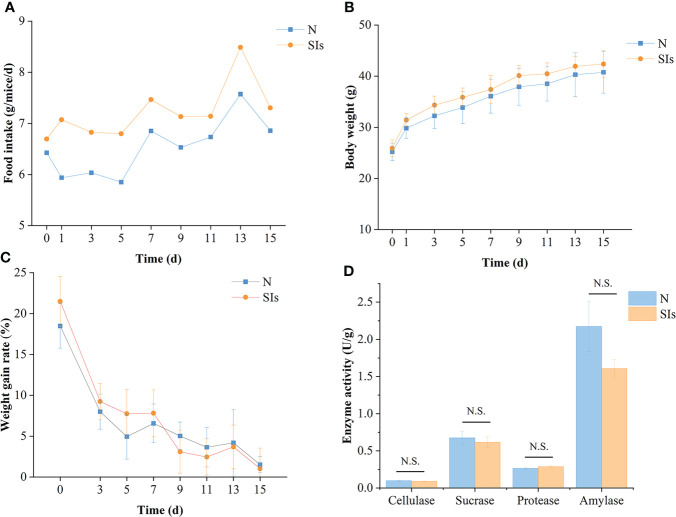
Effects of soybean isoflavones on the growth and digestive enzyme activities of normal mice. **(A)** Food intake of mice (*n* = 9). **(B)** Body weight of mice (*n* = 9). **(C)** Weight gain rate of mice (*n* = 9). Weight gain rate is the ratio of increment in weight and initial weight. **(D)** Digestive enzyme activities of mice (*n* = 3). N, normal group; SIs, soybean isoflavones group. In horizontal ordinate, 0 = the last day of acclimation. N.S., no significance (*P* > 0.05).

SIs disturbed the food intake of mice initially but returned to normal quickly (**Figure 2A**). In total, mice treated with SIs showed higher food intake than normal mice during the intervention phases (**Figure 2A**). The body weight of mice in the SIs group was higher than that in the N group during treatment, while the weight gain rate was lower than that in the N group from day 9 to 15 (**Figures 2B, C**). Although the weight gain rate was not statistically significant, SIs exhibited weight control effects.

### Effects of SIs on the digestive enzyme activity of normal mice

Enzymatic activity is involved in food metabolism. To evaluate the effect of SIs on digestive enzyme activity, the activities of cellulase, sucrase, protease, and amylase were measured. The results showed that the cellulase, sucrase, and amylase activities of the mice in the SIs group were lower, whereas the protease activity was higher than that in the N group (**Figure 2D**). However, there were no significant differences between the two groups. These results indicated that SIs modulated the digestive enzyme activity and further affected food metabolism.

### Effects of SIs on the gut microbiota of normal mice

**Figure 3:**
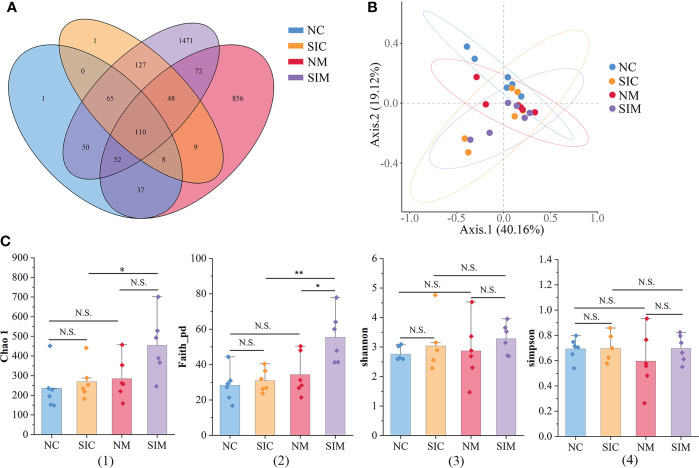
Effects of soybean isoflavones on the gut microbiota diversity of normal mice (*n* = 6). **(A)** Operational taxonomic unit (OTU) numbers of the gut microbiota of different groups. **(B)** Beta diversity of the gut microbiota (PCoA, Bray-Curtis distance). **(C)** Alpha diversity of the gut microbiota. (1) Chao 1, (2) Faith_pd, (3) Shannon, (4) Simpson. NC, lumen-associated bacteria of the normal group; SIC, lumen-associated bacteria of the soybean isoflavones group; NM, mucosa-associated bacteria of the normal group; SIM, mucosa-associated bacteria of the soybean isoflavones group. N.S., no significance (*P* > 0.05), ^*^ *P* < 0.05, ^**^ *P* < 0.01.

In this study, 1,684,265 demultiplexed sequences were obtained from raw data by barcode splitting, and 1,344,003 feature sequences were obtained after quality filtering and trimming, denoising, merging, and removing chimeric sequences (**Supplementary Table S2**). As shown in **Figure 3A**, more OTUs were found in the intestinal content and mucosa samples of the SIs group than in those of the N group. The PCoA results showed that LAB and MAB of mice in the two groups could be distinguished (**Figure 3B**). However, the samples in the SIs group were far from each other, suggesting that SIs changed the diversity and uniformity of the gut microbiota.

Alpha diversity indices (including Chao1, Faith_pd, Shannon, and Simpson) were used to evaluate the richness and/or evenness of the gut microbiota. The discrepancies of alpha diversity indices were compared between the N and SIs groups and between LAB and MAB. For both LAB and MAB, all alpha diversity indices in the SIs group were higher than those in the N group (**Figure 3C**). Among them, Faith_pd of MAB was significantly increased after treatment with SIs (*P* < 0.05), suggesting that SIs were beneficial to increase the microbial diversity of normal mice. All alpha diversity indices did not show significant differences between the LAB and MAB in the N group. Interestingly, higher levels of Chao1 (*P* < 0.05) and Faith_pd (*P* < 0.01) were observed in MAB than in LAB of mice treated with SIs, indicating that SIs intervention promoted more new bacteria growing in mucosa than in lumen. However, the abundance of these bacteria was very low.

**Figure 4:**
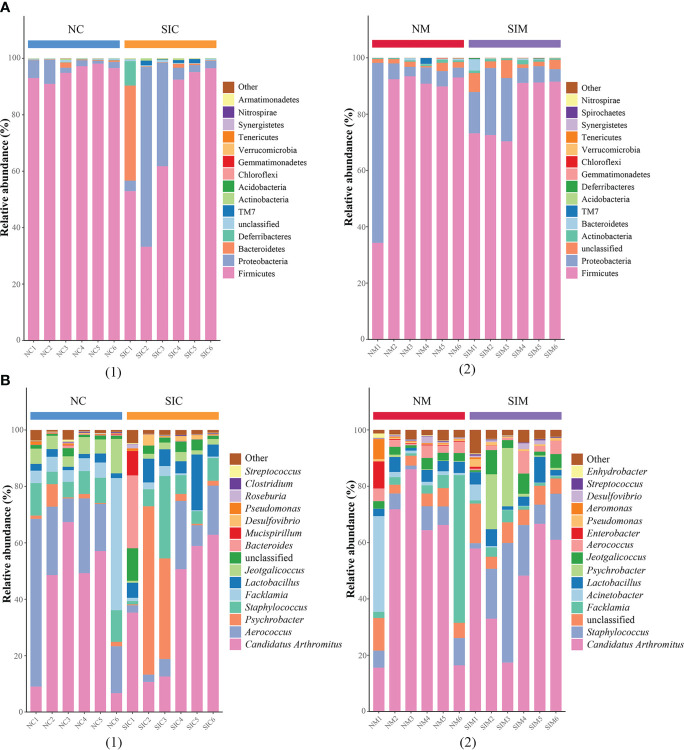
Effects of soybean isoflavones on the gut microbiota structure of normal mice at **(A)** phylum and **(B)** genus level (*n* = 6). (1) lumen-associated bacteria, (2) mucosa-associated bacteria. NC, lumen-associated bacteria of the normal group; SIC, lumen-associated bacteria of the soybean isoflavones group; NM, mucosa-associated bacteria of the normal group; SIM, mucosa-associated bacteria of the soybean isoflavones group.

**Figure 5:**
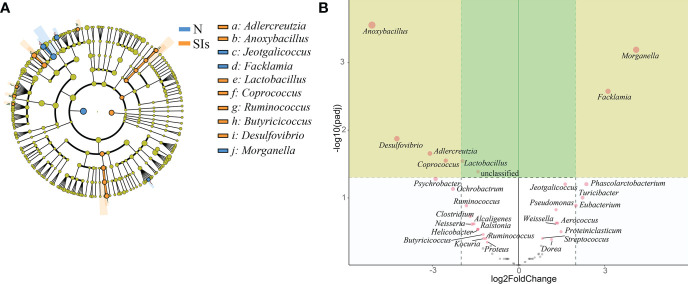
Significance analysis on operational taxonomic unit (OTU) difference of lumen-associated bacteria between groups. **(A)** Linear discriminant analysis effect size (LEfSe) analysis at the genus level (linear discriminant analysis [LDA] score > 4). **(B)** DESeq2 analysis at the genus level. N, normal group; SIs, soybean isoflavones group.

Firmicutes and Proteobacteria were the two dominant phyla in LAB and MAB in both groups (**Figure 4A**). SIs treatment decreased the relative abundance of Firmicutes while increasd the relative abundance of Bacteroidetes. As a result, the ratio of Firmicutes to Bacteroidetes (F/B) decreased in both LAB and MAB of the SIs group. At the genus level, *Candidatus arthromitus* was the dominant genus in the two groups, and it was slightly decreased in the SIs group for both LAB and MAB (**Figure 4B**). LEfSe analysis revealed more differential bacteria among LAB than among MAB. *Facklamia*, *Jeotgalicoccus*, and *Morganella* were the differential bacteria in the LAB of normal mice, and they significantly decreased after SIs treatment (**Figure 5A**). However, *Lactobacillus, Adlercreutzia, Coprococcus, Ruminococcus*, *Butyricicoccus*, and *Desulfovibrio* increased and became the differential bacteria in the LAB of the mice treated with SIs (**Figure 5B**). In MAB, *Acinetobacter* was the only differential bacterium in normal mice and was down-regulated by SIs (6.69 vs. 1.13%). Additionally, *Psychrobacter* was enriched in mice treated with SIs (0.26 vs. 7.09%). These results indicate that the effects of SIs on LAB were more profound than those on MAB.

### Effects of SIs on the SCFAs metabolism of normal mice

**Figure 6:**
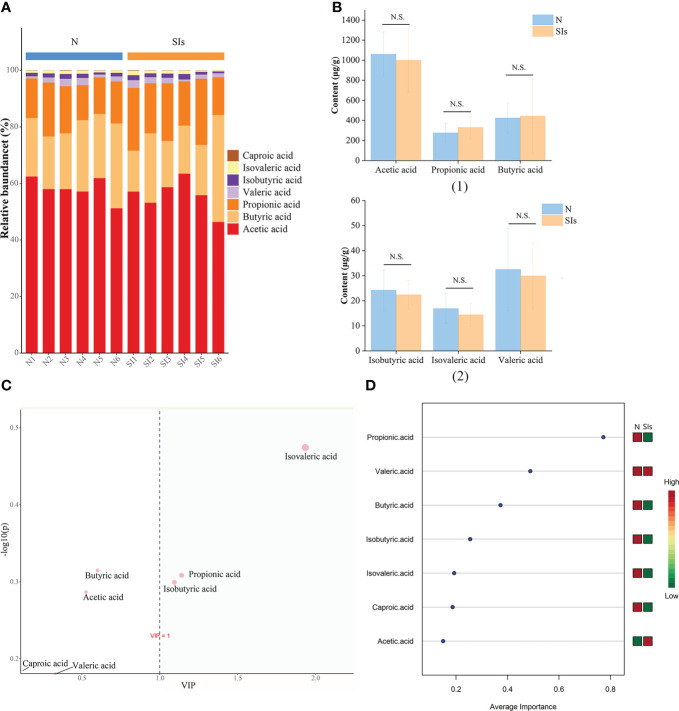
Effects of soybean isoflavones on short-chain fatty acids (SCFAs) of normal mice (*n* = 6). **(A)** The relative abundance difference of SCFAs between the two groups. **(B)** The content difference of SCFAs between the two groups. **(C)** Orthogonal partial least squares-discriminant analysis (OPLS-DA). **(D)** Support vector machines (SVM) analysis. N, normal group; SIs, soybean isoflavones group. N.S., no significance (P > 0.05).

SCFAs are the primary end-products of fermentation of non-digestible carbohydrates (Morrison and Preston, 2016). Thus, we detected the SCFAs contents in the colonic feces of mice after SIs intervention. The most common SCFAs were acetic acid, butyric acid, and propionic acid, which constitute 96% of SCFAs present in the mice colon in the N and SIs groups (**Figure 6A**). After SIs intervention, the acetic acid, valeric acid, isobutyric acid, isovaleric acid, and caproic acid contents decreased, while butyric acid and propionic acid contents increased (**Figure 6B**). To identify potential biomarkers in response to SIs treatment, OPLS-DA and SVM were used to analyze SCFAs data. Isovaleric acid, propionic acid, and isobutyric acid, with value importance in projection (VIP) > 1, were the biomarkers identified by OPLS-DA analysis (**Figure 6C**); propionic acid (average importance = 0.77) was the most important SCFA identified by SVM analysis (**Figure 6D**). Taken together, propionic acid may be a differential SCFA for distinguishing the N and SIs groups. Out of our expectations, all SCFAs showed no significant differences between the N and SIs groups.

**Figure 7:**
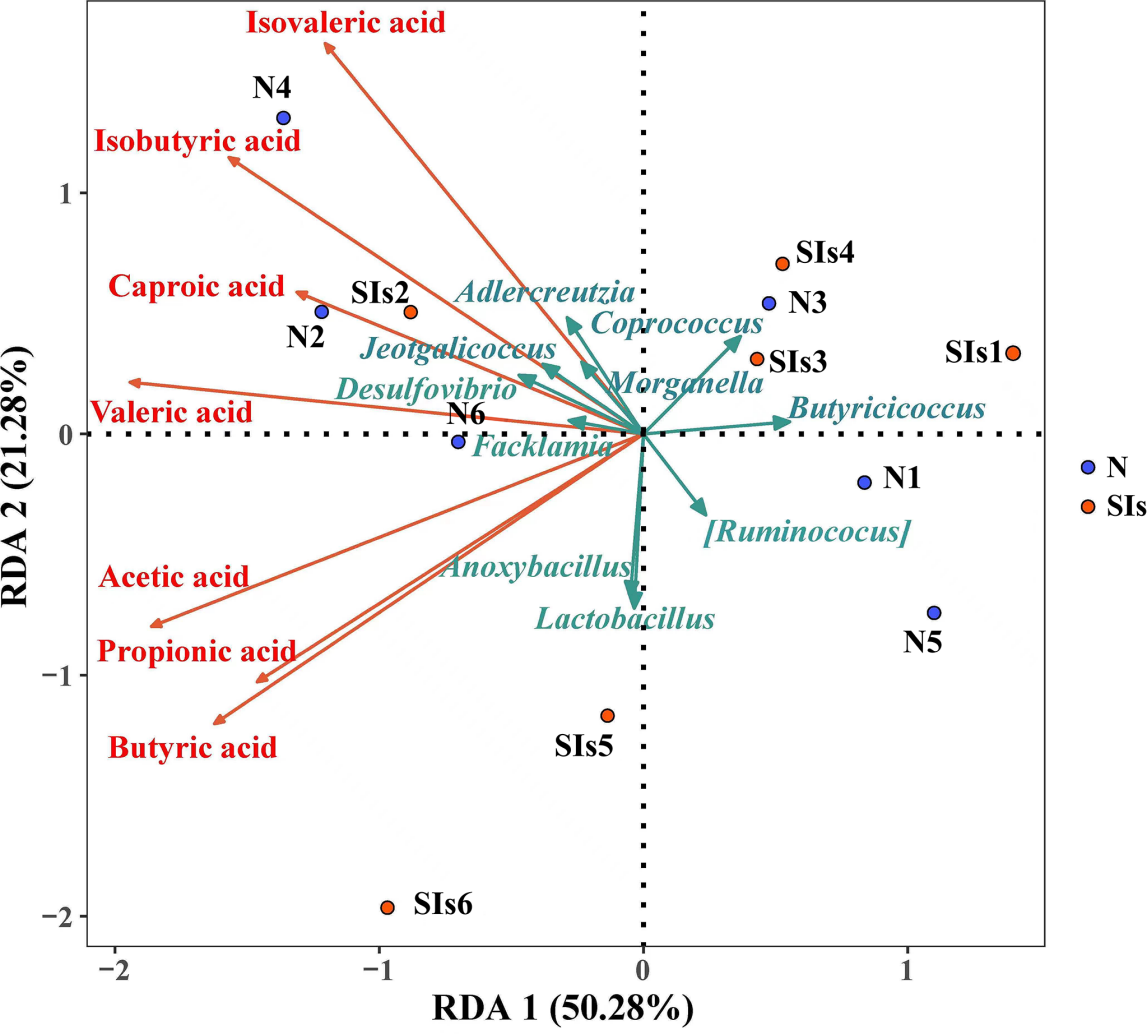
Correlation between the differential bacteria and short-chain fatty acids (SCFAs). (Redundancy analysis; *n* = 6). Colored arrows represent differential bacteria and SCFAs, colored dots represent samples from different groups. Angle between the arrow lines represented correlation; acute angle represents positive correlation and obtuse angle represents negative correlation. N, normal group; SIs, soybean isoflavones group.

SCFAs metabolism is the result of a complex interplay between diet and the gut microbiota (Morrison and Preston, 2016). RDA was used to explore the correlations between the gut microbiota and SCFAs metabolism. The results showed that acetic acid, propionic acid and butyric acid were positively, but caproic acid, isovaleric acid and isobutyric acid were negatively correlated with *Lactobacillus* (**Figure 7**). In addition, the relative abundance of *Anoxybacillus*, *Facklama*, *Desulfovibrio*, and *Jeotgalicoccus* was positively correlated with acetic acid, propionic acid, and butyric acid. *Adlercreutzia* was positively correlated with isovaleric acid, isobutyric acid, caproic acid, valeric acid, and acetic acid. These results suggested that these microbes were the key genera responding to SIs metabolism and affecting SCFAs formation.

## Discussion

“Let food be your medicine” was an ancient idea that is still popular in western countries today. Accordingly, “one root of medicine and food” is the guiding ideology of the Chinese people regarding health care. SIs are widely distributed in leguminous foods and medicinal and edible Chinese traditional medicines, and are commonly used to ameliorate menopause-associated obesity and metabolic dysfunction (Hirose et al., 2016). Thus, we were interested in the potential benefits of SIs in normal hosts. In this study, SIs intervention reduced the weight gain rate at a higher level of food intake, which indicates that SIs may also be beneficial for weight control in healthy hosts. Furthermore, the activities of cellulases, amylases, and sucrases were reduced after SIs intervention, which may result in the reduction of glucose produced by decomposition of the body and the absorption of glucose by fatty acids and complex carbohydrates in triglycerides. This process may be one of the mechanisms by which SIs control metabolic diseases such as obesity.

To further understand the mechanism underlying the weight control effect of SIs, 16S rRNA gene sequencing was used to clarify the changes in the gut microbiota of mice. It is important to note that the small intestine contains most of the gut receptors, and immune and nerve cells, and is increasingly implicated in microbe-host crosstalk (de Vos et al., 2022). The duodenum and its microorganisms are major factors in various metabolic and immune diseases (de Vos et al., 2022). Gut microbiota is composed of LAB and MAB with different ecological niches. LAB are mainly related to metabolism, whereas MAB are closely related to immunity (Zhang et al., 2021). To comprehensively analyze the effects of SIs on the gut microbiota, we observed changes in LAB and MAB after SIs treatment. The results showed that the structural changes in LAB were more significant than those in MAB, and more specific bacteria were produced. These results imply that SIs mainly affect the metabolic function of normal mice.

The Firmicutes/Bacteroidetes (F/B) ratio is regarded as an important indicator of intestinal homeostasis (Shin et al., 2015). It has been reported that a higher F/B ratio was usually observed in overweight and obese people and animals (Koliada et al., 2017; Abenavoli et al., 2019; Stojanov et al., 2020). In this study, a lower F/B ratio was observed after SIs treatment, which correlated with a decrease in Firmicutes and an increase in Bacteroidetes. Studies have revealed that Firmicutes possess higher metabolic diversity than Bacteroidetes, and are involved in 393 and 92 metabolic pathways, respectively (Caspi et al., 2020). Moreover, Firmicutes were more conducive to the fermentation and metabolism of carbohydrates and lipids than Bacteroidetes (Koliada et al., 2017; Stojanov et al., 2020). *Clostridium* and *Streptococcus*, two genera of the phylum Firmicutes, stimulate the secretion of cellulase and sucrase, respectively (Guo et al., 2019; Elsababty et al., 2022). The abundance of the two genera was reduced after SIs treatment, which may result in the decreased activities of cellulase and sucrase. Alterations in the gut microbiota can trigger changes in digestive enzyme activity, which further affects food metabolism.

It is worth noting that some beneficial bacteria, including *Lactobacillus*, *Adlercreutzia*, *Coprococcus*, *Ruminococcus*, and *Butyricicoccus*, are enriched after SIs intervention. Among them, the relative abundance of *Lactobacillus* was higher than that of the other genera in the SIs group. *Lactobacillus* is an essential probiotic that can prevent obesity by altering the gut microbiota in obese mice (Zhang et al., 2022). It has been reported that *Lactobacillus* can cut down sugars for energy and produce large amounts of lactic acid, which can increase satiety after a meal (Lv et al., 2022). Moreover, *Lactobacillus* converts glycosides into aglycones to improve their bioavailability and bioactivity by producing extracellular *β*-glucosidase. Liu et al. (2018) found that *Lactobacillus* and the pathway of glycan biosynthesis and metabolism decreased after intake of non-isoflavone diet. Therefore, it can be considered that *Lactobacillus* is a specific response bacterium in SIs-treated mice. In addition, *Adlercreutzia* plays an important role in glycolipid metabolism, and low abundance of *Adlercreutzia* is associated with glycolipid metabolism disorders (Lu et al., 2021). In this study, the relative abundance of *Adlercreutzia* was also increased in the mice treated with SIs. Thus, the beneficial effect of SIs on the metabolism of normal mice is related to enriched abundance of *Lactobacillus* and *Adlercreutzia*.

SCFAs play an important role in regulating glucose homeostasis, lipid metabolism, and appetite (Morrison and Preston, 2016). McNabney and Henagan (2017) demonstrated that SCFAs are potential metabolic targets for preventing and counteracting obesity. Gut microbiota metabolizes flavonoids to produce SCFAs (Li et al., 2022). Therefore, diet-driven changes in the gut microbiota led to variations in SCFAs (Morrison and Preston, 2016). Correlation analysis indicated that the relative abundance of *Lactobacillus* was positively correlated with acetic acid, butyric acid and propionic acid. Some *Lactobacillus* strains (*L. rhamnosus* GG, *L. gasseri* PA 16/8, *L. salivarius spp salcinius* JCM 1230, *L. agilis* JCM 1048, and *L. acidophilus* CRL 1014) participate in the production of acetic acid, propionic acid, and butyric acid (Markowiak-Kopeć and Śliżewska, 2020). *Coprococcus*, *Ruminococcus*, and *Butyricicoccus* are regarded as “anti-inflammatory” butyrate-producing bacteria. The abundance of *Coprococcus* was significantly positively correlated with the abundance of genes encoding the terminal step in butyrate formation (Altemani et al., 2021). Several studies (Liang et al., 2021; Lin et al., 2021) have shown that *Ruminococcus* can ferment complex sugars to produce acetic acid, propionic acid, and butyric acid. *Butyricicoccus* is considered a bacterium with prebiotic potential, which can consume acetate and produce butyrate as its main end product when grown on monosaccharides and disaccharides (Geirnaert et al., 2014; Trachsel et al., 2018). Butyrate has anti-inflammatory properties and can enhance the intestinal barrier function and mucosal immunity (Liu et al., 2018). Thus, the differential bacteria identified by LEfSe analysis were responsible for the changes of SCFAs profile. A study found that SIs increased butyric acid and acetic acid levels in rats (Luo et al., 2019). Our study showed butyric acid and propionic acid contents increased after SIs treatment. However, there was no significant difference in all types of SCFAs between normal mice and mice treated with SIs, which may be related to dose or intervention time.

The major limitations of this study are its dosage and intervention time design, which lead to incomprehensive results. Unlike medicine, the effect of foods and medicinal and edible Chinese traditional medicines is milder and thus more suitable for long-term health care. Therefore, further studies comparing different doses and intervention times are warranted.

## Conclusion

Dietary supplementation with SIs, even in the short term, may be beneficial for weight control, and the specific mechanism is related to regulating gut microbiota, changing digestive enzyme activities, and further affecting metabolism. Among these, *Lactobacillus*, *Adlercreutzia*, *Coprococcus*, *Ruminococcus*, and *Butyricicoccus* are the key beneficial genera that affect normal organisms. This study may improve the current understanding of the interaction between SIs, intestinal digestive enzymes, and microbiota, providing evidence for the use of SIs to prevent obesity.

## Data availability statement

The data presented in the study are deposited in the NCBI repository, accession number PRJNA854044.

## Ethics statement

The experiments were approved by the Animal Care and Use Committee of Hunan University of Chinese Medicine (LL2020102103).

## Author contributions

GX designed the study. LH wrote the manuscript. TZ, LH, and HH collected and analyzed the data. GX reviewed the manuscript. All authors contributed to the article and approved the submitted version.

## Funding

This work was supported by the National Natural Science Foundation of China (81804076), the Natural Science Foundation of Hunan Province (2020JJ5426).

## Conflict of interest

The authors declare that the research was conducted in the absence of any commercial or financial relationships that could be construed as a potential conflict of interest.

## Publisher’s note

All claims expressed in this article are solely those of the authors and do not necessarily represent those of their affiliated organizations, or those of the publisher, the editors and the reviewers. Any product that may be evaluated in this article, or claim that may be made by its manufacturer, is not guaranteed or endorsed by the publisher.

## Supplementary material

Supplementary Figure 1 The total ion chromatogram (TIC). **(A)** Mix standards; **(B)** Samples; **(C)** The overlap chromatogram of qualitiy control and sample.

Supplementary Figure 2 Quality assessment and control of short-chain fatty acids data using principal component analysis (PCA). **(A)** The score plot of the normal group; **(B)** The score plot of the soybean isoflavones group. The point falling outside the ellipse suggests that it may be an outlier.

Supplementary Figure 3 Data standardization of short-chain fatty acids. **(A)** Content distribution of each short-chain fatty acid before and after standardization. **(B)** Content distribution of each sample before and after standardization.

Supplementary Table 1 The methodological investigation of GC-MS.

Supplementary Table 2 The numbers of demultiplexed and feature sequences in each sample.
