# Supplementary figures and images for: Soybean isoflavones modulate gut microbiota to benefit the health weight and metabolism
Source: Front Cell Infect Microbiol. 2022 Sep 2;12:1004765. doi: 10.3389/fcimb.2022.1004765 (PMC9478439; doi:10.3389/fcimb.2022.1004765)

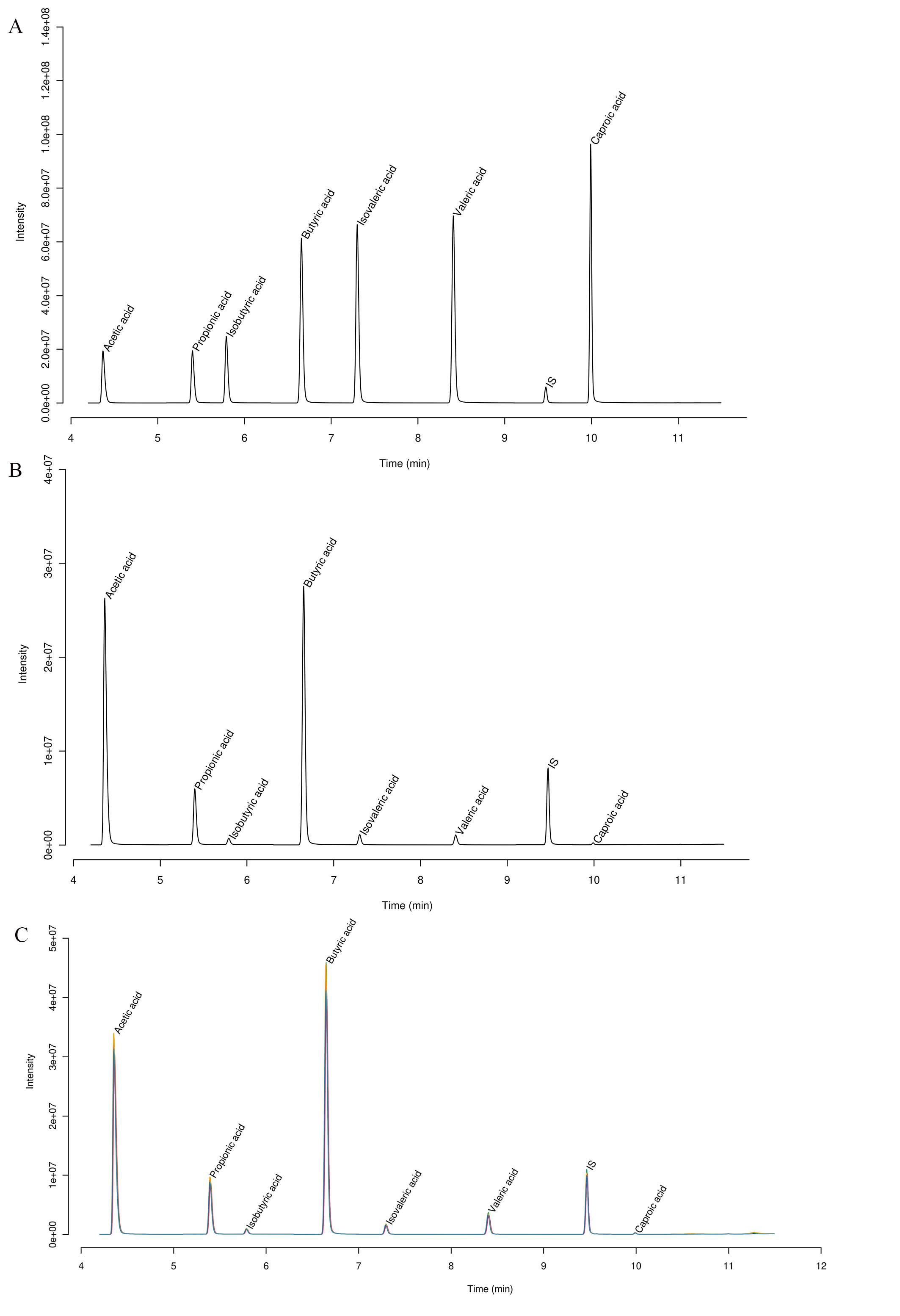

Supplement: Supplementary Figure 1 — The total ion chromatogram (TIC). (A) Mix standards; (B) Samples; (C) The overlap chromatogram of qualitiy control and sample. [file Image_1.tif]

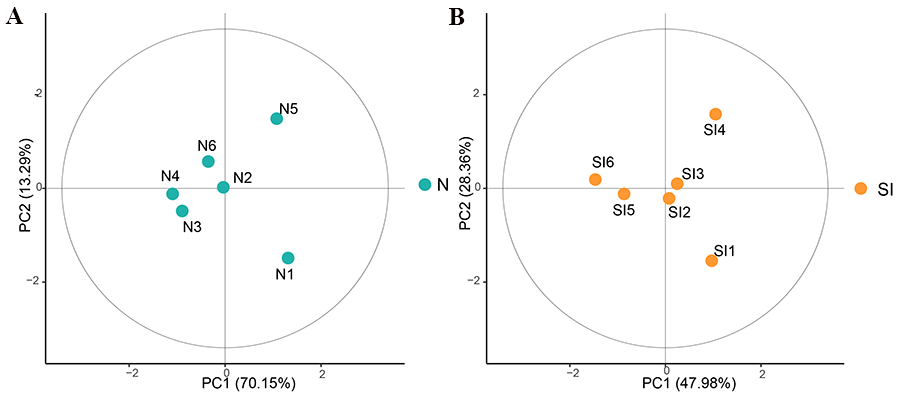

Supplement: Supplementary Figure 2 — Quality assessment and control of short-chain fatty acids data using principal component analysis (PCA). (A) The score plot of the normal group; (B) The score plot of the soybean isoflavones group. The point falling outside the ellipse suggests that it may be an outlier. [file Image_2.tif]

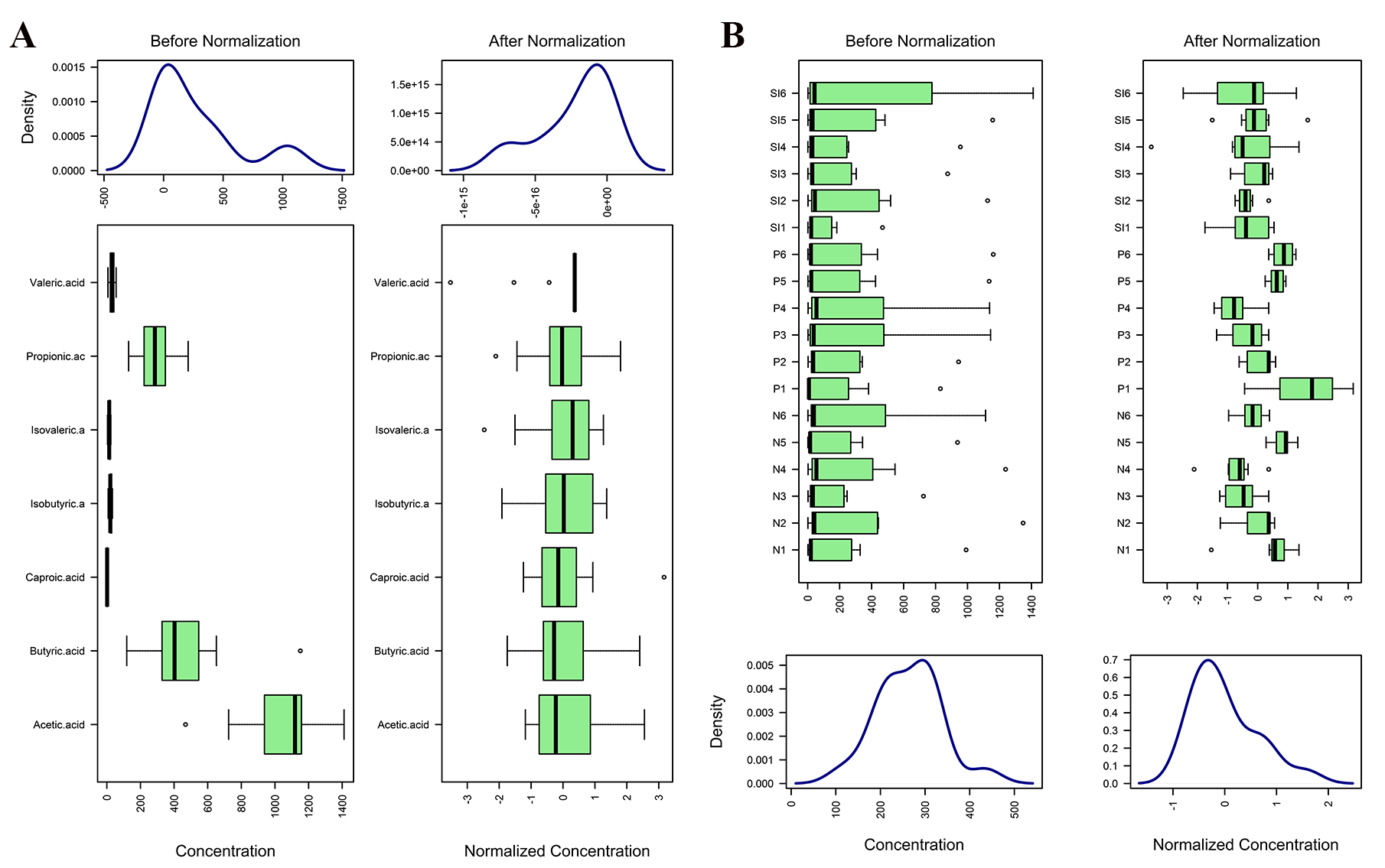

Supplement: Supplementary Figure 3 — Data standardization of short-chain fatty acids. (A) Content distribution of each short-chain fatty acid before and after standardization. (B) Content distribution of each sample before and after standardization. [file Image_3.tif]
