# Supplementary material for: Soybean isoflavones modulate gut microbiota to benefit the health weight and metabolism
Source: Front Cell Infect Microbiol. 2022 Sep 2;12:1004765. doi: 10.3389/fcimb.2022.1004765 (PMC9478439; doi:10.3389/fcimb.2022.1004765)
Supplement: Supplementary Table 1 — The methodological investigation of GC-MS. [file Table_1.pdf]

Table S1 The methodological investigation of GC-MS

standards	t_R (min)	linear regression equations	correlation coefficient (r)	linearity of calibration (μg/mL)	intra-day precision RSD (%)	inter-day precision RSD (%)	repeatability RSD (%)	recovery (%)
acetic acid	4.37	y=0.0393x+0.0154	0.9903	0.02-500	1.85	11.73	5.44	90.34-106.31
propionic acid	5.40	y=0.0382x+0.0002	0.9947	0.02-500	1.73	5.28	4.72	87.53-94.55
isobutyric acid	5.79	y=0.0475x+0.0004	0.9953	0.02-500	1.31	4.76	5.65	85.20-107.07
butyric acid	6.66	y=0.1170x+0.0009	0.9955	0.02-500	1.57	6.87	5.51	85.56-96.35
isovaleric acid	7.30	y=0.1265x+0.0005	0.9956	0.02-500	1.35	6.63	7.59	85.50-92.82
valeric acid	8.41	y=0.1334x+0.0002	0.9955	0.02-500	1.30	5.66	7.68	88.16-99.25
caproic acid	9.99	y=0.1209x+0.0023	0.9938	0.02-500	1.00	7.71	9.53	85.22-96.97
