# Supplementary material for: Soybean isoflavones modulate gut microbiota to benefit the health weight and metabolism
Source: Front Cell Infect Microbiol. 2022 Sep 2;12:1004765. doi: 10.3389/fcimb.2022.1004765 (PMC9478439; doi:10.3389/fcimb.2022.1004765)
Supplement: Supplementary Table 2 — The numbers of demultiplexed and feature sequences in each sample. [file Table_2.pdf]

Table S2 The numbers of demultiplexed and feature sequences in each sample

sample	demultiplexed sequences	filtered	denoised	merged	Non-chimeric	Percentage of input non-chimeric
NC1	75797	69284	67904	66236	59977	79.13
NC2	69726	63680	62410	61127	55291	79.30
NC3	72334	67080	64433	62126	58716	81.17
NC4	73673	67416	66556	65314	60136	81.63
NC5	69722	63681	61972	60446	57371	82.29
NC6	71190	65686	63974	62835	57586	80.89
NM1	62305	51987	50504	49076	44204	70.95
NM2	65520	59867	58155	56908	54091	82.56
NM3	62144	54733	52982	51423	50717	81.61
NM4	79222	71942	70532	68678	62371	78.73
NM5	68671	61374	58880	56600	54662	79.60
NM6	67869	59671	56446	54817	53036	78.14
SIC1	71956	67530	65784	63486	60619	84.24
SIC2	70625	61927	59851	58173	55555	78.66
SIC3	70344	62537	58957	56836	51944	73.84
SIC4	68627	61766	59950	58307	55046	80.21
SIC5	74995	68370	67201	65629	62654	83.54
SIC6	71353	64671	63040	61571	57000	79.88
SIM1	69235	64251	61134	57796	55344	79.94
SIM2	69461	62803	58449	56361	52961	76.25
SIM3	64531	54739	52010	50581	48951	75.86
SIM4	72146	67451	64789	62734	59394	82.32
SIM5	67649	62139	60393	58253	55588	82.17
SIM6	75170	68835	66895	65633	60789	80.87
Total	1684265	1523420	1473201	1430946	1344003	-
Mean	70177.7	63475.8	61383.4	59622.8	56000.1	79.74
